# Anemia associated with asymptomatic malaria among pregnant women in the rural surroundings of Arba Minch Town, South Ethiopia

**DOI:** 10.1186/s13104-015-1081-4

**Published:** 2015-03-31

**Authors:** Desalegn Nega, Daniel Dana, Tamirat Tefera, Teferi Eshetu

**Affiliations:** Malaria and Other Parasitic and Vector-Borne Diseases Research Team, Directorate Of Parasitic, Bacterial and Zoonotic Diseases Research, Ethiopian Public Health Institute, P.O. Box 1242/5654, Addis Ababa, Ethiopia; Department of Medical Laboratory Sciences and Pathology, College of Public Health and Medical Sciences, Jimma University, P.O. Box 378, Jimma, Ethiopia

**Keywords:** Anemia, Asymptomatic malaria, Blood smear microscopy, Arbaminch, Ethiopia

## Abstract

**Background:**

Anemia during pregnancy is a well known medical condition most of the time under-recognized as it is overshadowed by the normal physiological condition during pregnancy. This study aimed at determining the prevalence and predictors of anemia among pregnant women residing in the rural surroundings of Arbaminch Town, south Ethiopia.

**Methods:**

A cross-sectional community based study was conducted between April and June, 2013. A structured questionnaire was used to collect socio-economic and socio-demographic characteristics of the pregnant women. Hematocrit (HCT) level was determined to classify the pregnant women as anemic and non-anemic. Diagnosis of asymptomatic malaria parasitemia was done by Giemsa stained blood smear microscopy. HCT < 33%, (HCT ≥ 30% & < 33%), (HCT ≥ 21% & < 30%), and HCT < 21% was used to indicate anemia, mild anemia, moderate anemia, and severe anemia respectively.

**Results:**

A total of 341 pregnant women participated in this study, out of which 118 (34.6%) were anemic. The median age of the pregnant women was 25 years (Inter-quartile range: 23–29). The mean HCT was 35.2% (95% CI: 34.6%–35.8%) with SD of ±5.5%. Of those 118 anemic women; 73(61.9%) were mildly anemic, 38(32.2%) were moderately anemic, and 7(5.9%) were found to be severely anemic. The prevalence of asymptomatic malaria parasitemia was 9.1% (31/341). The odds of being anemic were 15.72 times [AOR: 15.72, 95% CI (3.97, 62.22), P-value ≤ 0.001] more likely to occur in parasitemic individuals relative to the non parasitemic pregnant women. Not using insecticide treated bed net (ITN) was a significant predictor of anemia among the pregnant women [AOR: 3, 95% CI: (1.72, 5.22), P < 0.001].

**Conclusion:**

This study highlighted the significant association between anemia and asymptomatic malaria among pregnant women in the study area. Therefore, the practice of routine screening for malaria and anemia followed by prompt management should be encouraged to curb the effect of malaria and anemia on the pregnant women as well as her fetus. Further studies should also be in place to test the effectiveness of routine screening for malaria and anemia followed by prompt management.

## Background

Anemia, defined as the reduction of the number of red blood cells with a consequent decrease in their oxygen-carrying capacity, is one of the major public health problems affecting the health and socio-economy of both the developed and developing countries [1].

Although iron deficiency is the principal cause of anemia, parasitic infection such as malaria, schistosomiasis and hookworm disease, other nutritional deficiencies, and haemoglobinopathies are also responsible [2].

Globally, anemia affects 1.62 billion people which correspond to 24.8% of the population. The global prevalence of anemia among pregnant women is estimated to be 41.8% and majority of them live in Africa, where more than 17 million pregnant women are known to be affected [1].

It is generally established that women demonstrate an increased prevalence and severity of malarial infection during pregnancy [3,4]. In areas where there is high malaria transmission, infected individuals tend to remain asymptomatic as a result of acquired immunity [4,5].

Anemia during pregnancy is a well known medical condition most of the time under-recognized as it is overshadowed by the normal physiological condition during pregnancy. However, studies have shown that asymptomatic infection with *Plasmodium* parasites is associated with anemia among pregnant women [5-7]. Maternal anemia consequently increases the incidence of maternal mortality and is associated with increased fetal and infant mortality, prematurity and low birth weight [8,9].

Although the complete mechanism of anemia associated with malaria is not understood, it was suggested that it involves the removal of red blood cells (erythrophagocytosis) from circulation as well as dyserythropoesis. It was also noted that the release of different cytokines in response to infection and malaria toxins also disturb erythropoesis [10-12].

In Ethiopia, over the last decade there is no significant change in the prevalence of anemia in pregnancy. According to the national demographic and health survey, the prevalence of anemia in pregnancy declined from 33.1% in 2005 to 29.9% in 2011 [13]. In this country, a special attention is given to the reduction of maternal mortality as to achieve the millennium development goal. Therefore, this study was conducted to provide reliable data on the burden of anemia among pregnant women and also indicated the importance of considering asymptomatic malaria in the elimination of malaria from the country.

## Methods

### Study area

This study was conducted in the rural surroundings of Arba Minch Town. The area, locally called as “Arba Minch Zuria Woreda”, is one of the Districts in the Southern Nations, Nationalities, and Peoples' Region of Ethiopia. Arba Minch is the capital of Gamo Gofa Zone which is located 505 km South of Addis Ababa, the capital city of Ethiopia. The villages are found at an average altitude of 1,206 m above sea level with a rainfall of 950 mm and with the same average annual temperature to the town.

### Study design

A community based cross sectional survey was conducted among apparently healthy pregnant women. Pregnant women with absence of disease symptom/sign within the past 48 hours, axillary temperature ≤ 37.5°c, permanent residents in the study area, and those willing to participate in the study and signed the informed consent were included. Individuals having taken anti-malarial drugs in the past six weeks prior to data collection, those undergoing any kind of long term medical treatments, and unwilling individuals were excluded from the study.

### Sample size and sampling technique

The required sample size for this study was calculated using a formula for a single population proportion. Taking expected prevalence of anemia as 29.9% [13], 95% confidence level and 5% margin of error, the calculated sample size was 322. By taking non-response rate of 10%, the total sample size became 354.

Multistage sampling technique was employed in selection of the study subjects. Eight Study villages were selected by simple random sampling using the lottery method. The sample size was then distributed proportionally to the villages based on the size of their pregnant women population. The selected study villages were: Chano Mile, Chano Dorga, Chamo Shele, Chano Chalba, Aelgo Gonto, Genta Kenchama, Chano Lante, and Kolla Shara.

At the village level, the households were selected by simple random sampling by using the sampling frame which was prepared after having identified the pregnant women in the households by the preliminary assessment through active house-to-house visits. Computer generated random numbers were used for the random selection of the study households or study participants.

### Data collection

A pre-tested semi-structured questionnaire was administered by trained interviewer to obtain information on socio demographic characteristics of the study participants. Capillary blood samples were collected by a finger pricking using disposable lancet following aseptic technique. The blood filled hematocrit tubes sealed with a sealing-wax were placed in a labeled larger tube container and then transported to Arba Minch University Hospiatal.

### Laboratory investigations

Hematocrit level was determined by using microhematocrit centrifuge as described elsewhere [14]. The hematocrit level was read by using a Hawksleys microhematocrit reader. According to WHO guidelines, pregnant women are normal with hematocrit concentration of 33% and above. The pregnant women with HCT values less than 33% were categorized as anemic. Anemic women were further categorized as women with mild anemia (HCT ≥ 30% & < 33%), moderate anemia (HCT ≥ 21% & < 30%) and severe anemia (HCT below 21%) [14].

Thick and thin blood smears stained with Giemsa stain were examined under light microscope with 1000x magnification. Parasite density was determined as described elsewhere [15].

### Statistical analysis

Data were coded, entered into, cleaned and analyzed using SPSS for windows version 16.0. Both descriptive and inferential statistics were employed for the analysis of data. Binary logistic regression was employed to assess the predictors of anemia. Variables significant at P-value of 0.25 in the univariate logistic regression were selected for Multivariate logistic regression analysis model. Odds ratios with 95% confidence intervals were calculated and p < 0.05 values were considered to be statistically significant.

### Ethical considerations

The study protocol was reviewed and approved by the ethical review board of Jimma University. Community agreement and local oral consent was obtained from community leaders. Written informed consent was obtained from all of the study participants. Data collected from each study participant and results of laboratory tests were kept confidential and used only for the research purpose. Result of participants with parasitic infection was addressed to the study participants. The pregnant women who were found to be infected with *Plasmodium* parasite were referred for treatment and medical consultation in the ANCs of nearby health facilities.

## Results

### Socio-demographic characteristics of the pregnant women

A total of 341 pregnant women agreed to participate in the study and provided blood specimen and necessary information. The age of the study participants ranged from 17 to 40 years with a median age of 25 years old (Inter-quartile range: 23–29). Majority of the pregnant women belonged to the age group of 21–25 years (41%). Most of them were house wives (39%), completed a primary education (39%), multigravidae (44%), in their second trimester (41%), attending their ANC follow-up (80.6%), and use ITN always (84.8%) (Table 1).

**Table 1:** **Sociodemographic and economic characteristics of the pregnant women in the rural surroundings of Arba Minch, Town South Ethiopia, April to June, 2013**

**Variables**	**No. (%)**	**Variables**	**No. (%)**
**Age groups**		**Marital status**	
≤20	32(9.4)	married	327(95.9)
21-25	140(41.1)	single	4(1.2)
26-30	102(29.9)	divorced	6(1.8)
31-35	47(13.8)	widowed	4(1.2)
>35	20(5.9)		
**Parity**		**Gestational age**	
Primigravidae	91(26.7)	1^st^ trimester	83(24.3)
Secondgravida	99(29.0)	2^nd^ trimester	140(41.1)
Multigravida	151(44.3)	3^rd^ trimester	118(34.6)
**Occupation**		**Education**	
Farmer	98(28.7)	Illiterate	59(17.3)
Daily laborer	40(11.7)	Read/write	15(4.4)
Merchant	57(16.7)	Primary	134(39.3)
House wife	134(39.3)	Secondary	95(27.9)
Civil servant	12(3.5)	College/above	38(11.1)
**Family size**		**ANC Attendance**	
1-3	116(34.0)	Yes	275(80.6)
4-7	168(49.3)	No	66(19.4)
>7	57(16.7)		
**Income**		**ITN use**	
<650 ETB	204(59.8)	Use always	187(54.8)
650-1300 ETB	102(29.9)	Use rarely	19(5.6)
>1300 ETB	35(10.3)	Do not use	135(39.6)
**Religion**		**IRS(past 12 months)**	
protestant	227(66.6%)	Yes	289(84.8)
orthodox	114(33.4%)	No	52(15.2)

### Prevalence of anemia and associated factors

Of the total 341 study participants, 118 (34.6%) were anemic. The minimum and maximum HCT values were 18% and 46%, respectively, while the mean was 35.2% (95% CI: 34.6%–35.8%) with SD of ±5.5%. Of those 118 anemic women; 73(61.9%) were mildly anemic, 38(32.2%) were moderately anemic, and 7(5.9%) were found to be severely anemic (Table 2).

**Table 2:** **Multivariate logistic regression analysis of predictors for anemia among pregnant woman in rural surroundings of Arbaminch town, Southern Ethiopia, April to June 2013**

**Variables**	**No. examined**	**No. anemic (%) 118 (34.6%)**	**COR (95% CI)**	**p-value**	**AOR (95% CI)**	**p-value**
**Age**						
≤20	32	11(34.4)	0.35(0.11, 1.11)	0.074	0.17(0.01, 5.94)	0.327
21-25	140	41(29.3)	0.28(0.11, 0.73)	0.009*	0.70(0.09, 5.42)	0.734
26-30	102	31(30.4)	0.29(0.11, 0.78)	0.014*	0.61(0.08, 4.60)	0.632
31-35	47	23(48.9)	0.64(0.22, 1.85)	0.408	0.83(0.23, 3.02)	0.774
≥36	20	12(60.0)	1.00		1.00	
**Education**						
Illiterate	59	30(50.8)	3.88(1.53, 9.85)	0.004*	2.45(0.43, 14.04)	0.316
Read/write	15	7(46.7)	3.28(0.91, 11.80)	0.069	2.14(0.27, 17.19)	0.475
Primary	134	49(36.6)	2.16(0.92, 5.10)	0.077	1.52(0.30, 7.78)	
Secondary	95	24(25.3)	1.27(0.51, 3.14)	0.608	0.71(0.13, 3.90)	0.618
College/above	38	8(21.1)	1.00		1.00	0.692
**Income**						
<650 ETB	204	82(40.2)	3.01(1.38, 6.58)	0.003	2.15(0.65, 7.05)	0.207
650-1300 ETB	102	32(31.4)	1.97(0.92, 4.22)	0.027	1.51(0.49, 4.68)	0.476
>1300 ETB	35	4(11.4)	1.00		1.00	
**Parasitemia**						
Positive	31	28(90.3)	22.8(6.7, 76.9)	<0.001*	15.72(3.97, 62.22)	<0.001**
Negative	310	90(29.0)	1.00		1.00	
**Gestational age**						
1^st^ trimester	83	28(33.7)	1.26(0.69, 2.30)	0.457	0.86(0.36, 2.02)	0.721
2^nd^ trimester	140	56(40.0)	1.647(0.98, 2.78)	0.061	2.03(1.06, 3.86)	0.031**
3^rd^ trimester	118	34(28.8)	1.00		1.00	
**ITN use**						
Use always	187	38(20.3)	1.00		1.00	
Use sometimes	19	12(63.2)	2.72(2.48, 18.23)	<0.001*	1.96(1.82, 19.47)	0.001**
Do not use at all	135	68(50.4)	3.98(2.44, 6.50)	<0.001*	2.99(1.72, 5.22)	0.001**
**IRS (In past 12 months)**						
Yes	289	94(32.5)	1.00		1.00	
No	52	24(46.2)	1.78(0.98, 3.23)	0.059	0.80(0.32, 2.03)	0.661
**Present ANC follow-up**						
Yes	275	83 (30.2%)	1.00		1.00	
No	66	35(53%)	2.61(1.51, 4.51)	0.001*	3.39(1.49, 7.69)	0.004**

*significant at p < 0.25; hence considered for multivariate analysis.

**significant at p <0.05.

All of the study participants were screened for *Plasmodium* parasites by using Giemsa stained blood film. It was found that the prevalence of asymptomatic plasmodium infection was 9.1% (31/341). The species diagnosed include *P. falciparum* (38.71%), *P. vivax* (48.38%)*, and* mixed infection (12.9%).

More than 90% of parasitemic individuals were anemic while only 29% of non-parasitemic individuals had anemia. The odds of being anemic was 15.7 times [AOR: 15.7 2, 95% CI (15.723.97, 62.22), P-value ≤ 0.001] more likely to occur in parasitemic individuals relative to the non-parasitemic pregnant women (Table 2). However, parasite density was not associated with severity of anemia (*X*^2^ = 3.7, p = 0.157).

Fifty point eight percent (30/59) of the pregnant women who were illiterate were found to be anemic while only 21.1% (8/38) of individuals who had tertiary level of education were anemic. However, the difference was not statistically significant [AOR: 2.45, 95% CI (0.43, 14.04)].

The prevalence of anemia was 34.1%, 33.3%, and 35.8% among primigravidae, secundigravidae, and multigravidae respectively. There was no statistically significant association between gravidity and anemia among the pregnant women (*X*^2^ = 0.17, p = 0.918).

The prevalence of anemia significantly differed between the different gestational ages. Forty percent (56/140) of individuals in their second trimester were anemic while 28.8% (34/118) of individuals in their third trimester were anemic. The difference was statistically significant [AOR: 2.03, 95% CI (1.06, 3.86)] table 2.

Another factor which was associated with anemia among the pregnant women was the use of ITN. The results of the study revealed that the odds of being anemic was almost 2 fold [(95% CI: 1.82-19.47), P = 0.003] among rare ITN users, and 3 fold [(95% CI: 1.72-5.22), P < 0.001] among those who did not use ITN completely relative to those who were using ITN always.

More than half (53%) of individuals who did not attend ANC were anemic while 30.2% of those who attended ANC were anemic. The odds of being anemic was more than three times among those who did not attend ANC as compared to those who attended ANC [AOR: 3.39, 95% CI (1.49, 7.69)].

Pearson Correlation coefficient was calculated to figure out correlation between HCT value and the continuous variables (age, parity, parasite density, family size, and family income). The results of the study revealed that the increase in malaria parasite density was significantly correlated with a decrease in HCT values (r = −0.463, P = 0.009). Parity was also negatively correlated with HCT though the correlation was not statistically significant (r = −0.017, p = 0.758). Mothers’ age (r = −0.187, p = 0.001) and family size (r = −0.124, p = 0.022) were negatively correlated with HCT value. On the other hand, family income was positively correlated with HCT (r = 0.115, p = 0.035).

## Discussion

The present study attempted to assess the association of asymptomatic *plasmodium* infection with the prevalence of anemia among pregnant women in the rural surroundings of Arbaminch Town, Ethiopia. The prevalence of anemia among the pregnant women was determined to be 34.6%, which was by far lower than the findings of studies conducted elsewhere [5-7,16-18]. The contrast might be due to differences in socio-demography, socio-economy, and altitude. The nutritional status, infections with soil transmitted helminths, HIV, and malaria will greatly contribute to anemia [17,19,20].

Asymptomatic malaria was significantly associated with anemia among the pregnant women. This is in agreement with the findings of other studies reported elsewhere [5,21]. In this study, an increase in malaria parasite density was significantly correlated with a decrease in HCT values. This result is supported by the fact that the higher the number of parasites the higher the number of infected red blood cells and the higher the number of destroyed red blood cells [22].

Age was negatively correlated with hematocrit of the pregnant women. This is in line with other studies conducted elsewhere [23,24]. However, it was in contrast with the findings of a study conducted in Trinidad and Tobego [25]. The discrepancy might be due to the difference in the socio-demographic and economic characteristics, geographical location, and nutritional status of the study participants.

The findings of the present study showed that ANC attendance had a protective effect against anemia. This might be due to the provision of iron and folic acid supplements during ANC visits [26]. This highlights the significance of advocating ANC follow-up among pregnant women.

In the present study, the use of ITN had a protective effect against anemia. This might be as a result of the protective effect of ITN against malaria and consequently against anemia. The findings of other studies also supported our result [27,28]. A study in Malawi also indicated the association between absence of bed net and lower hemoglobin levels [29].

The findings of the present study showed that family size was negatively correlated with the level of hematocrit. This might be due to the fact that as the size of a family increases unless there is subsequent increase in income the capacity to get balanced diet would also decrease. On the other hand, the family income was positively correlated with the hematocrit level.

The present study had its own limitations; confounding factors such as the nutritional status of individuals, Iron and folic acid supplementation during the course of pregnancy, infection with soil-transmitted helminths, HIV, and tuberculosis were not assessed to rule out the independent effect of asymptomatic malaria on anemia among the pregnant women. In addition the study design, which is a cross-sectional study, suffers from the lack of identifying the causal relationships. The wide range of confidence intervals shows that the sample size was not sufficient. Nevertheless, the multi-stage community based nature of this study assures the representativeness of the study.

## Conclusion

In conclusion, the prevalence of anemia among the pregnant women was 34.6% (118/341). Of those 118 anemic women; 73(61.9%) were mildly anemic, 38(32.2%) were moderately anemic, and 7(5.9%) were found to be severely anemic. The prevalence of asymptomatic malaria parasitemia was 9.1% (31/341). There was a significant association between anemia and asymptomatic malaria parasitemia. Thus, the practice of routine screening for malaria and anemia followed by prompt management should be encouraged to curb the effect of malaria and anemia on the pregnant women as well as her fetus. Furthermore, a study designed to test the effectiveness of the practice of routine screening for malaria and anemia followed by prompt management is also needed.

## References

[1] World Health Organization. Worldwide prevalence of anaemia 1993–2005: WHO global database on anaemia / Edited by Bruno de Benoist. Ines Egli and Mary Cogswell: Erin McLean; 2008.

[2] Hisano M, Suzuki R, Sago H, Murashima A, Yamaguchi K (2010). Vitamin B6 deficiency and anaemia in pregnancy. Eur J Clin Nutr..

[3] Brabin BJ (1983). An analysis of malaria in pregnancy in Africa. Bull World Health Organ.

[4] WHO. Malaria in pregnancy: guidelines for measuring key monitoring and evaluation indicators. In: World health organization 2007. 2007.

[5] Matangila JR, Lufuluabo J, Ibalanky AL, Luz RA, Lutumba P, Geertruyden JPV (2014). Asymptomatic *Plasmodium falciparum* infection is associated with anemia in pregnancy and can be more cost-effectively detected by rapid diagnostic test than by microscopy in Kinshasa Democratic Republic of the Congo. Malar J..

[6] Ekanem EI, Agan TU, Efiok EE, Ekott MI, Okodi E (2010). A study of anemia in women with asymptomatic malaria parasitaemia at their first antenatal care visit at the General Hospital, Ikot Ekpene, Akwa Ibom State Nigeria. Asian Pac J Trop Med.

[7] Douamba Z, Bisseye C, Djigma FW, Compaor´e TR, Bazie VJT, Pietra V (2012). Asymptomatic malaria correlates with anemia in pregnant women at Ouagadougou, Burkina Faso. J Biomed Biotechnol.

[8] Menendez C (1995). Malaria during pregnancy: a priority area of malaria research and control. Parasitol Today..

[9] Leopardi O, Naughten W, Salvia L, Colecchia M, Matteelli A, Zucchi A (1996). Malaric placentas. A quantitative study and clinico-pathological correlations. Pathol Res Pract.

[10] Haldar K, Mohandas N (2009). Malaria, erythrocytic infection, and anemia. Hematology Am Soc Hematol Educ Program.

[11] Jelkmann W, Pagel H, Wolff M, Fandrey J (1992). Monokines inhibiting erythropoietin production in human hepatoma cultures and in isolated perfused rat kidneys. Life Sci..

[12] Faquin WC, Schneider TJ, Goldberg MA (1992). Effect of inflammatory cytokines on hypoxia-induced erythropoietin production. Blood..

[13] UNFPA. Trends in maternal health in Ethiopia. Challenges in achieving the MDG for maternal mortality. In: In-depth analysisof the EDHS 2000–2011. Ethiopia: Addis Ababa; 2012.

[14] WHO. Recommended method for determination of packed cell volume by centrifugation prepared by Expert Panel on Cytometry of International Council for Standardization in haematology Document WHO. 2000. p. DIL 00 2 1 9.

[15] Nwaghaa UI, Ugwub VO, Nwaghac TU, Anyaehied BU (2009). Asymptomatic plasmodium parasitaemia in pregnant Nigerian women: almost a decade after roll back malaria. Trans R Soc Trop Med Hyg J..

[16] Getachew M, Yewhalaw D, Tafess K, Getachew Y, Zeynudin A (2012). Anaemia and associated risk factors among pregnant women in Gilgel Gibe dam area. Southwest Ethiopia Parasites and Vectors..

[17] Agan TU, Ekabua JE, Udoh AE, Ekanem EI, Efiok EE, Mgbekem MA (2010). Prevalence of anemia in women with asymptomatic malaria parasitemia at first antenatal care visit at the University of Calabar Teaching Hospital, Calabar, Nigeria. Int J Women’s Health.

[18] Nwali MI, Umeora OUJ, Ozumba BC, Onoh RC, Ezeonu PO, Agwu UM (2014). Anemia among unbooked parturients with asymptomatic malaria parasitemia at a tertiary institution southeast Nigeria. IOSR Journal of Dental and Medical Sciences.

[19] Ogunbode O, Okonofua F, Odunsi K (2003). Anaemia in pregnancy. Contemporary obstetrics and gynecology for developing countries.

[20] Lamina MA, Sorunmu TO (2003). Prevalence of anaemia in pregnant women attending the antenatal clinic in a Nigerian university teaching hospital. Nigerian Med Pract..

[21] Adam I, Khamis AH, Elbashir MI (2005). prevalence and risk factors for anemia in pregnant women of eastern Sudan. Trans R Soc Trop Med Hyg.

[22] Oni GA, Oguntibeju OO (2006). Relationships between the malaria parasite density and children anaemia: brief communication. Int J Pattern Recognit Artif Intell.

[23] Ania BJ, Suman VJ, Fairbanks VF, Melton LJ (1994). Prevalence of anemia in medical practice: community versus referral patients. Mayo Clin Proc..

[24] Salive ME, Cornoni-Huntley J, Guralnik JM, Phillips CL, Wallace RB, Ostfeld AM (1992). Anemia and hemoglobin levels in older persons: relationship with age, gender, and health status. J Am Geriatr Soc..

[25] Uche-Nwachi EO, Odekunle A, Jacinto S, Burnett M, Clapperton M, David Y (2010). Anaemia in pregnancy: associations with parity, abortions and child spacing in primary healthcare clinic attendees in Trinidad and Tobago. Afr Health Sci.

[26] Abdullahi H, Gasim GI, Saeed A, Imam AM, Adam I (2014). Antenatal iron and folic acid supplementation use by pregnant women in Khartoum Sudan. BMC Res Notes..

[27] Hawley WA, Phillips-Howard PA, Kuile FO, Terlouw DJ, Vulule JM, Ombok M (2003). Community-wide effects of permethrin-treated bed nets on child mortality and malaria morbidity in western Kenya. Am J Trop Med Hyg..

[28] Abdulla S, Armstrong-Schellenberg J, Nathan R, Mukasa O, Marchant T, Smith T (2001). Impact on malaria morbidity of a programme supplying insecticide treated nets in children aged under 2 years in Tanzania: community cross sectional study. BMJ..

[29] Holtz TH, Marum LH, Mkandala C, Chizani N, Roberts JM, Macheso A (2002). Insecticide-treated bednet use, anaemia, and malaria parasitaemia in Blantyre District Malawi. Trop Med Int Health.

